# The Multidimensional Assessment of Interoceptive Awareness (MAIA)

**DOI:** 10.1371/journal.pone.0048230

**Published:** 2012-11-01

**Authors:** Wolf E. Mehling, Cynthia Price, Jennifer J. Daubenmier, Mike Acree, Elizabeth Bartmess, Anita Stewart

**Affiliations:** 1 University of California San Francisco, Osher Center for Integrative Medicine, San Francisco, California, United States of America; 2 University of Washington, Biobehavioral Nursing and Health Systems, Seattle, Washington, United States of America; 3 University of California San Francisco, Center for AIDS Prevention Studies, San Francisco, California, United States of America; 4 University of California San Francisco, Institute for Health & Aging, Center for Aging in Diverse Communities, San Francisco, California, United States of America; Royal Holloway, University of London, United Kingdom

## Abstract

This paper describes the development of a multidimensional self-report measure of interoceptive body awareness. The systematic mixed-methods process involved reviewing the current literature, specifying a multidimensional conceptual framework, evaluating prior instruments, developing items, and analyzing focus group responses to scale items by instructors and patients of body awareness-enhancing therapies. Following refinement by cognitive testing, items were field-tested in students and instructors of mind-body approaches. Final item selection was achieved by submitting the field test data to an iterative process using multiple validation methods, including exploratory cluster and confirmatory factor analyses, comparison between known groups, and correlations with established measures of related constructs. The resulting 32-item multidimensional instrument assesses eight concepts. The psychometric properties of these final scales suggest that the Multidimensional Assessment of Interoceptive Awareness (MAIA) may serve as a starting point for research and further collaborative refinement.

## Introduction

Terms such as *body awareness*, *somatic awareness*, or *interoceptive awareness* are used in many different ways in medicine, psychology, neuroscience, anthropology, philosophy, and popular discourse, often without precision or distinctive definitions, and generally with discipline-specific meanings and implications. Definitions for interoception may differ, for example, between psychophysiologists and neuroscientists. We attempt to provide more clarity for these constructs by integrating viewpoints and language from the multiple disciplines, for which mind-body processes and the interaction of mind and biology have become major research topics. This paper describes the systematic development of a new self-report instrument for these constructs.

Starting from a health science and clinical practice background with a particular interest in integrative pain management, we found that contradictory views exist in Western medicine regarding the value of body awareness. Much of the earlier literature considers a patient’s attentional focus on body symptoms as an expression of anxiety, depression, or somatization [1]. For example, the terms *body awareness* and *somatic awareness* have been used in studies of anxiety and panic disorders to describe a cognitive attitude characterized by an exaggerated focus on physical symptoms, magnification (“somatosensory amplification”), rumination, and catastrophic outcome beliefs [2]. Consequently, the numbers of perceived and presumed potentially distressing body sensations have served as markers for anxiety and somatization [2], and somatic or body awareness has commonly been viewed as maladaptive. (As the terms *body awareness* and *somatic awareness* are essentially synonymous, we will use only the simpler term *body awareness*.).

More recently, an alternate view of body awareness as potentially beneficial for health has emerged [3], for example, the ability to recognize subtle body cues [1], and accordingly a number of therapeutic approaches now aim deliberately to enhance body awareness. Clinical research has suggested health benefits of body awareness for patients with a variety of diagnoses (for a review see [4], [5]). Proponents of the body awareness construct as beneficial for health usually refer to a particular kind of awareness characterized by *mindfulness*, nonjudgmental acceptance, and a sense of self grounded in experiencing physical sensations in the present moment, sometimes summarized as a sense of embodiment [6]–[8].

By differentiating aspects of body awareness, such as different modes of attention towards body sensations, we may be able to understand contradictory views of body awareness. Whether body awareness is beneficial or maladaptive may depend on “distinct and incompatible modes of mind” [9]–[11] associated with brain functions that are habitually integrated but may be uncoupled after, for example, a few weeks of meditation [12]. Focusing attention directly on immediately experienced feelings appears to be adaptive, whereas an abstract ruminative self-focus appears to be maladaptive [13]. Learning to regulate one’s attention in specific ways may be a key feature of body awareness-enhancing practices and, therefore, a dimension to be differentiated within the body awareness construct. Similarly, enhanced body awareness by means of a specific form of attention regulation training has been used in a therapeutic approach to phantom pain. This training is termed “concrete somatic monitoring” or “sensory discrimination” [14] of the detailed characteristics of physical sensations as opposed to a rather diffuse, emotion-based vigilance [2], [15].

These findings and notions imply that different modes of attention and variations in the ability to regulate attention may explain seemingly contradictory interpretations of body awareness. A more differentiated view may help to overcome the ambiguity of the body awareness construct by discerning multiple dimensions within the construct, such as modes of attention [4], and relating these to established concepts in the biomedical literature, namely to proprioception, interoception, and mindfulness.

For the biomedical literature, a neuroscientific and physiological understanding of body awareness would presumably entail both proprioceptive and interoceptive awareness. Although most proprioceptive and interoceptive perception remains unconscious, *proprioceptive awareness* refers to the conscious perception of joint angles and muscle tensions, of movement, posture, and balance [16]; *interoceptive awareness* refers to the conscious perception of sensations from inside the body that create the sense of the physiological condition of the body, such as heart beat, respiration, satiety, and the autonomic nervous system sensations related to emotions [17]–[20]. The term *interoception* was introduced 1906 by Sherrington [21] and has had its own history of definitions, at times including proprioception [19] or suggesting its inclusion [17], while other times it was clearly separated from proprioception as visceral perception. On the basis of newer neuroanatomy research, Craig redefined interoception as the sense of the physiological condition of the material body [18], which includes autonomic sensory nerve input from the entire body as well as pain and sensuous touch and is neuroanatomically distinct from proprioception.

Within the fast-growing literature on interoception, a body of research is emerging that links awareness of all internal physical sensations to regional brain activities, specifically in the somatotopically organized anterior insular cortex. These insula activities appear to provide a multilevel integrated metarepresentation of the state of the entire body and include the inner-body experience of emotions and pain [22]. It has been experimentally demonstrated that the link between interoceptive awareness and physical sensations (e.g., of emotions) is a key element for affect regulation [23], [24], decision making [23], [25], and for the sense of self [26]–[29]. Interindividual variations in interoceptive capacity have been found to be associated with right anterior insula cortical thickness, suggesting potential neuroplasticity effects of interoceptive awareness [30], [31], an interpretation further supported by recent longitudinal studies of a mindfulness-based stress reduction intervention [32], [33]. Much of this research is related to interoceptive awareness as a key element in meditation and stress reduction [34]–[36] and has become the subject of increasing research activities in recent years [23], [30], [37]–[42].

Although this research has led to a new understanding of how emotions [27], [40], [43]–[46] and the perception of pain [47], [48] are related to interoception, it has to a large degree stayed away from key behavioral and cognitive aspects well-known in perception and psychological pain research, such as appraisal and beliefs (e.g. catastrophizing), attention regulation (e.g. ignoring, distraction), behavior (e.g. avoidance, coping), anticipation, and past experience. Leading neuroscientific models of emotion and interoception only tangentially mention these psychological aspects as attribution processes [49]. Yet, interoceptive awareness is a product of conscious perception, and as such is a psychobiological process that is modified by complex bidirectional interactive evaluative functions, which are influenced by appraisal, beliefs, past experience, expectations, and contexts. Like the psychophysiologist Cameron [50] and others [51], we propose to broaden the conceptualization of interoceptive awareness as commonly used in neuroscience to one that includes these interpretational and organizing aspects of perception.

In summary, a more complex, multidimensional view of body awareness has emerged in recent years, which distinguishes modes of attention such as thinking about the body and presence in the body. The human capacity to move from thinking about physical symptoms (interpreting, appraising, and eventually ruminating with fearful hypervigilance) to a state of perceptual presence within the body, often labeled as *mindfulness* [9], [52], [53], is both the subject of philosophical discourse and a particular quality of body awareness [54]–[56]. Reflecting the complexity of the construct, Mehling et al. operationally defined body awareness as the sensory awareness that originates from the body’s physiological states, processes (including pain and emotion), and actions (including movement), and functions as an interactive process that includes a person’s appraisal and is shaped by attitudes, beliefs, and experience in their social and cultural context [4]. Dimensions of critical importance have been laid out in [4], [5]. This conceptualization encompasses both proprioceptive and interoceptive awareness from psycho-physiological as well as neuroscientific viewpoints, is biologically based on proprioceptive and interoceptive neural activity, and includes well-established cognitive and behavioral aspects of perception.

Considering the potential clinical importance of the construct, particularly as a mediator of therapies for painful conditions, very few attempts have been made to date to measure body awareness, including whether it changes in response to therapies claiming to enhance it [57]; and even fewer attempts have been made to link intervention-related changes in body awareness to clinical outcomes [58].

Objective measures for the accuracy of proprioceptive and interoceptive awareness have been increasingly developed and applied in recent years. Proprioceptive awareness has been studied by objective measures, such as joint repositioning angles or biofeedback devices, and applied in research on Tai Chi [59], [60] and yoga [61], but not on meditation. Objective measures for interoceptive awareness have been widely used in an organ-specific fashion with heart-rate detection accuracy tasks, respiratory resistance threshold detection and discrimination tasks, and the detection of intestinal stimuli. However, none of these has been shown sensitive to changes by body awareness-enhancing approaches, with the exception of the heart rate detection task in meditators when subjected to dramatic arousal by intravenous infusions with adrenaline [62], [63]. So far it is unclear whether these organ-specific methods are appropriate to show training-related changes in interoceptive or body awareness [64]. Interoceptive afferents within unimodular sensory systems are centrally integrated into a larger neural system that has been termed the homeostatic interoceptive system [18], [65], and preliminary studies support the notion that interoceptive awareness may reflect a general sensitivity for visceral processes with trait and state aspects that covary across modalities [66]–[68]. Objective measures allow for experimental studies, but are restricted to laboratory settings and reflect singular aspects of a person's complex experience.

A recent review of existing body awareness questionnaires and their psychometric properties showed that most questionnaires were based on the earlier conceptualization of body awareness as proxy measures for anxiety, lacked systematic development, were unidimensional, and missed key domains that might help discern between adaptive and maladaptive aspects of body awareness [4]. Commonly used measures of the closely related mindfulness construct include a much broader awareness focus on thoughts and exteroceptive stimuli and lack a more specific sensory focus on inner body sensations.

**Figure 1:**
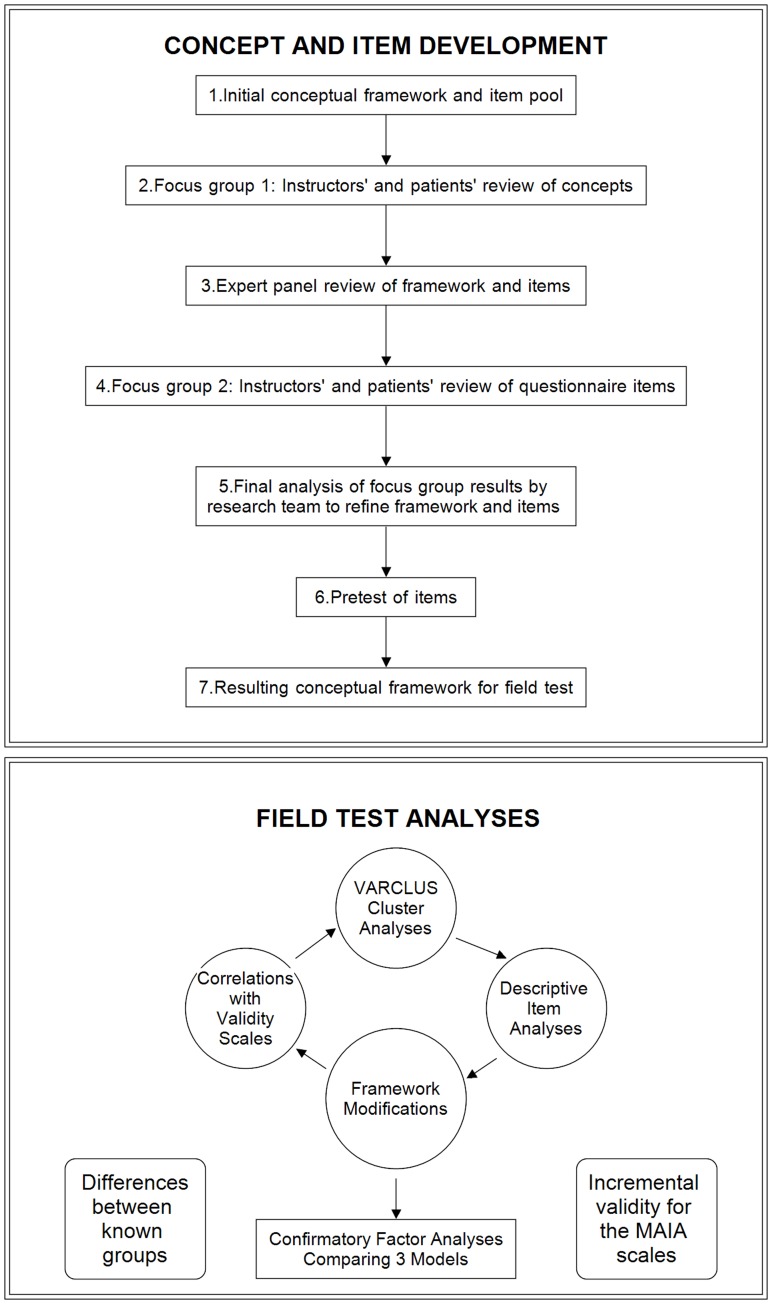
Iterative Sequence of Development and Testing of the MAIA.

Therefore, we used a mixed methods approach to systematically develop a self-report instrument for experimental interoception research and for assessment of mind-body therapies. The study and all procedures were approved by the university’s Institutional Review Board. The paper is organized into six main parts: (a) Concept and Item Development, (b) Field Test, (c, d, and e) three Construct Validity sections, and (f) Overall Discussion. A figure depicting the sequence is provided for ease of understanding the complexity of the approach (Figure 1). Because of our iterative mixed-methods approach [69], the concepts evolved during scale development. Thus this paper describes modifications to the conceptual framework throughout the process.

## Part 1: Concept and Item Development

As the common view of interoceptive awareness rarely includes any aspects of interpretation or organization of perception, and as no general consensus exists in the scientific literature regarding the body awareness construct, the first phase was to refine the multidimensional conceptual framework and develop a set of items reflecting its dimensions. Development of this framework and items was an iterative process involving six steps: (a) initial conceptual framework and item pool, (b) focus groups of instructors and patients to review concepts, (c) expert panel review of concepts and items, (d) second focus group with instructors and patients to review concepts and items, (e) analysis of all results, revision of framework and items by research team, and (f) pretest of items and analysis to prepare for field testing.

### 1. Initial Conceptual Framework and Item Pool

The initial conceptual framework had previously been developed in an iterative process [4] and included four dimensions with subdimensions. In their sequential order, these may be viewed as developmental qualities associated with ascending levels of body awareness [5]. As it can be expected that each dimension would correlate differently with other psychological constructs, measuring these dimensions reliably and separately may enhance our understanding of (a) processes of mind-body interaction (e.g. which dimensions affect pain perception), (b) variations in aspects of body awareness among individuals and groups, and (c) the appropriateness of targeted therapeutic interventions.

1. The awareness of body sensations includes the ability to identify inner sensations and to discern subtle bodily cues indicating varying functional states of the body and the emotional/physiological state. This dimension was seen as the primary sensory, physiological aspect of body awareness. Four subdomains were distinguished: (a) sensations of distress, worry, pain, and tension; (b) sensations of well-being; (c) neutral or ambiguous sensations; and (d) an affective aspect of these sensations, such as bothersomeness. The affect component of a body sensation was understood as resulting from early preconscious [70], [71] or secondary, evaluative appraisal [72].
2. As discussed above, the quality of attention was seen as a key dimension that should enable us to distinguish beneficial and maladaptive forms of body awareness It was differentiated into three subdomains: (a) The intensity of attention along a bipolar continuum – ranging from paying attention to sensations (an active response to the perception of sensations) on one end to distracted avoidance, ignoring and suppression of perceptions, on the other end - is a key factor, e.g., in the perception of pain sensations [73]–[75]. (b) The self-efficacy of attentional control, or the individual’s confidence in the ability to focus on a sensation and sustain or control the mode of attention, is increasingly studied with mind-wandering, and it can be improved by mind-body interventions [76], [77]. (c) The mode of attention describes how an individual pays attention to a sensation, whether her attention is more in a mode of (i) thinking about, reflecting on, judging, analyzing her sensation, with the extreme of ruminating, or (ii) nonjudgmental, immediate experience and sensory awareness of that sensation, with mindful presence as the polar opposite to rumination. This second dimension reflects a process component of body awareness, the active act of paying attention, which modifies, filters, or augments the sensory input from the body and is related to the broader concept of mindfulness.
3. The attitude of interoceptive awareness refers to two domains that describe how individuals relate to bodily cues. (a) Trusting or viewing bodily sensations as helpful for decision-making is an important component of chronic pain management [78] and the sense of self [18]. (b) Worry and catastrophizing about bodily cues are well-known major psychological attitudes modifying the perception, e.g., of pain [79]–[81]. This dimension was understood as a general trait-like bias towards appraisal of the perceived sensation and a modifier of the perceived sensations, a second key trait, relatively stable but potentially modifiable by targeted therapeutic interventions. Its effect on perceived sensations was thought to be mediated by the mode of attention (2c) [82], [83].
4. Mind-body integration was viewed as a goal inherent in mind-body therapies that can be experienced in two subdomains: (a) as emotional awareness, the awareness that certain physical sensations are the sensory aspect of emotions (as in the theory of “somatic markers” [27], [84]); or (b) as an overall felt sense of an “embodied self,” representing a second-order perception of sensations that contains within it a felt sense of the interconnectedness of mental, emotional, and physical processes as opposed to a disembodied sense of alienation and of being disconnected from one’s body [26], [85], [86].

Using this framework as well as recent literature and investigator clinical experience, we compiled an initial item pool of 306 items from twelve full and 10 partial body-awareness-relevant scales and subscales reviewed previously [4].

### 2. Focus Group 1: Instructors’ and Patients’ Review of Concepts

We conducted focus groups to obtain input from leading senior instructors of body awareness therapies, including mindfulness meditation, yoga, Tai Chi, Feldenkraïs, Alexander technique, breath therapy, and Somatic Experiencing, and from patients who received treatment from these instructors. Instructor and patient focus groups were held separately; both instructors and patients were recruited to attend two focus groups, the first to review and develop the concepts (FG-1), and the second to review items in relation to the concepts (FG-2). All focus group sessions were moderated by an independent professional moderator, digitally recorded, and transcribed verbatim. Details on recruitment and content are described elsewhere [5].

In the first set of focus groups (FG-1), the conceptual framework (defined above) was provided for reference (see details in [4]). The discussion focused on which dimensions were considered most important for their practice, and whether any dimensions were missing or needed modification.

In FG-1 analysis, a table was organized by dimensions and subdimensions, including new subdimensions identified in FG-1. For each subdimension, items from the original item pool and new items generated by the focus groups were listed along with relevant instructor and patient phrases. The research team reviewed these and modified the conceptual framework and items (see details in [87]). The result was a revised conceptual framework and a list of 67 items from older instruments and 101 items or item stems from patients and instructors, organized by concepts or dimensions.

### 3. Expert Panel Review of Framework and Items

The study team and a group of invited experts held an all-day conference to further refine the conceptual framework, review and rearrange items based on how well they fit with the revised conceptual framework, and create additional items.

Prior to the meeting, the expert group was sent the conceptual framework and the items including the patient and instructor quotes with guidelines for selecting the five best items from each dimension, and for modifying items or writing new ones as necessary: (a) item language should be such that it would make sense to everyone, including body-work-naïve individuals; (b) items should be able to capture any changes that might happen in individuals who receive training in these modalities; (c) breath should be reflected in each dimension if possible, as improving breath awareness was uniformly seen by practitioners in the first focus group as a key element of all approaches [5]; (d) items should reflect positive, negative, and neutral sensations; and (e) positively worded items were preferred.

Examples of the topics discussed at the meeting were conceptual distinctions among catastrophizing, distracting, ruminating, suppressing, and avoidance; thinking vs. feeling; whether to split directing attention from sustaining attention; distinction between ability to feel sensations and capacity to use that information as a behavior; whether trusting versus catastrophizing or controlling versus allowing are separate dimension; how to write items that are relevant to totally naïve respondents and social desirability issues. At the end of the meeting, the group reached consensus as to whether the structure and definitions in the revised conceptual framework adequately reflected the various issues discussed. After the meeting, the research team reviewed all comments and the revised framework and organized the 121 remaining and new items according to the new framework.

### 4. Focus Group 2: Instructors’ and Patients’ Review of Questionnaire Items

The goal of the second set of focus groups (FG-2), with instructors first and patients thereafter, was to review the revised conceptual framework and item pool, to improve item language and create new items.

*Instructor FG-2:* Prior to the meeting, the revised framework and items were mailed to participants with a request to rate each item using 0–5 structured response choices according to (a) how relevant the item was to its hypothesized dimension, and (b) how well the item could capture changes as a result of their particular therapeutic modality. They also were asked to cross out or revise any items they did not like, write new items, or suggest moving an item to another dimension. At the meeting, for each dimension, individual items favored by 3 or more instructors were discussed as to how well they reflected the dimension, and how well these items captured any changes that might occur in practice.

After the meeting, each instructor’s rating of relevance and likelihood to change was tabulated and summarized in a table, including open-ended suggestions. Items with clearly poor ratings were dropped, and items with the best ratings were highlighted. New items suggested by instructors were added for a total of 133 items. The research team reviewed the instructors’ item ratings and comments and dropped items that were considered redundant or problematic, or that were uniformly rejected. The team created a revised conceptual framework and a set of 119 items for review by the patients.

*Patient FG-2:* Prior to the meeting, participants were mailed the revised framework and items and asked to review the items, revise or delete items, and write new ones. In addition, they were asked to individually rate each item using structured responses ranging from 0 (useless question) to 5 (perfect question) for how well the item captured what they had learned from practicing their respective method. During the meeting, each dimension and the items with the highest ratings were discussed by the group and new items were created.

### 5. Final Analysis of Focus Group Results by Research Team to Refine Framework and Items

The research team synthesized the results from the focus groups and each patient’s item ratings and open-ended suggestions, and revised items based on the patients’ comments. Using a structured rating form, each team member individually indicated for each item whether it should be included in the next phase (cognitive pretest) and suggested alternative item wordings. A summary of these suggestions used by the team, along with guidelines from the earlier expert panel (Section 3 above) to finalize a set of 66 items for pretesting. Probes were designed for items that were potentially confusing, difficult to understand, or otherwise problematic.

Multiple options for format, instructions, and response scales were reviewed at this time. After selecting 3–4 possible response choice sets on a 0–5 response scale, we created sample “questionnaires” with the different options and their respective instructions. We decided to pretest the items using a format in which respondents would rate “how true” an item was for them. However, after a few pretests, this proved to be difficult, so we changed to “how often” with endpoints labeled 0 = *never* and 5 = *always*.

### 6. Pretest of Items

Cognitive interview testing refers to in-depth interviews designed to assess respondents’ understanding of questions and specific terms, and to identify difficulties with the response choices. We designed the pretest to consist of standard administration of all 66 items followed by in-depth probes for a subset of 28 items considered likely to be misinterpreted or otherwise problematic on the basis of patient and instructor ratings and comments. Probes were developed to determine whether respondents understood the intended meaning of specific words or phrases; whether similar questions were perceived as redundant; whether questions were offensive; to identify the cognitive processes used in responding; and to describe examples from respondents’ experience. For example, the item “I notice when I am uncomfortable in my body” was probed to query “What does the phrase ‘uncomfortable in your body’ mean to you?” Most probes depended on the response to the item.

We recruited a convenience sample of 16 patients and staff from the University Medical Center and the School of Medicine and of individuals known to the study team, including 6 patients with chronic pain, 5 “body awareness-naïve” and 5 “body awareness-experienced” staff. The pretest sample was primarily female (15/16), age ranged from 23 to 72 (mean = 44), education ranged from high school only to graduate school (4 high school or some college, 10 college degree, 2 post-graduate). Pretest interviews were audio recorded.

All pretest data including responses to probes were summarized for the 66 items, including information on the distribution of responses to each item. Seven items were dropped, and several items were revised or split into two items, resulting in 63 items retained for the field test.

### 7. Resulting Conceptual Framework for Field Test

The result of this iterative process was the following multidimensional conceptual framework, with 5 overarching dimensions and a total of 13 subdimensions. It reflects a slight modification of the initial framework described in Part 1.1.

1. Awareness of body sensations includes awareness of negative, positive, and neutral sensations, with no subdimensions or distinction as to whether these are perceived actively or passively. Sensations of breath were added as neutral sensations. Items from the original subdimension of affect were moved to become a subdimension of Emotional Reaction to Bodily Sensations.
2. Emotional reaction and attentional response to sensations includes four subdomains: (a) the affective response to a sensation, expressed as its bothersomeness or pleasantness (moved from Dimension 1); (b) suppressing, ignoring, or avoiding perceptions of sensations such as by distracting oneself; (c) narrative, judgmental awareness that “analyzes” sensation, including worrying that something is wrong; and (d) present-moment awareness with nonjudgmental awareness of sensations, i.e., a mindful presence. This reflects a substantial refinement of the original Dimension 2 labeled Quality of Attention.
3. Capacity to regulate attention pertains to various ways of controlling one’s attention as an active regulatory process. These include the ability to (a) sustain awareness, (b) actively direct attention to various parts of the body, (c) narrow or widen the focus of attention, and (d) allow sensations without trying to change them. This is a new dimension based on splitting the original “quality of attention.” Attentional control was originally a subdimension of Dimension 2, Quality of Attention, and now distinguishes several ways in which one can control attention.
4. Trusting body sensations, beliefs about importance of sensations reflects the extent to which one views awareness of bodily sensations as helpful for decision making or health. This single dimension was developed during the focus groups from items pulled from the original Dimensions 3 (Attitude of Interoceptive Awareness) and 4.
5. Mind-body integration (original Dimension 4) is viewed as the ultimate goal of mind-body therapies and includes three subdimensions: (a) emotional awareness, the awareness that certain physical sensations are the sensory aspect of emotions; (b) self-regulation of emotions, sensations, and behavior (developed in the focus groups); and (c) ability to feel a sense of an embodied self, representing a sense of the interconnectedness of mental, emotional, and physical processes as opposed to a disembodied sense of alienation and of being disconnected from one's body [26], [85], [86].

## Part 2: Field Test

Phase 2 was to conduct a field test of the preliminary survey and conduct psychometric analyses to identify a final set of scales. We describe here the survey and methods of data collection, the sampling and recruitment methods, and methods of analysis. Results of the field test are then described, including model testing and the final scales, internal consistency reliability, descriptive statistics, and scale-scale intercorrelations.

Respondents completed a one-time self-administered online survey using Survey Gizmo [88]. The survey consisted of 63 items assessing the concepts described above, demographic questions, and measures of related constructs.

### Methods: Participants

#### Eligibility

Our goal was to sample students and instructors experienced with at least one of the following therapies that include body awareness components: meditation/mindfulness, yoga, Tai Chi, Feldenkraïs Method, Alexander Technique, Breath Therapy, Massage (as professional training or practice only), or body-oriented psychotherapy (including Somatic Experiencing, Hakomi, and Rosen). To be eligible, the minimum requirement was at least 20 hours of exposure to formal training/instruction/therapy sessions.

#### Sampling design

To obtain variability in the sample, to examine whether there is a “developmental” aspect to learning these skills, and to conduct known-groups validity analyses by comparing means across experience levels, we aimed for about half of the sample to comprise individuals with extensive experience and half with less experience. We defined *highly experienced* as instructors with at least 5 years teaching experience in a mind-body therapy, and *less experienced* as students with at least 20 hours formal training/experience or instructors with less than 5 years of teaching experience in the mind-body therapy they were most familiar with. We aimed for a balance across the categories of therapies.

To identify those with extensive and less experience, we decided to recruit people from all of the types of therapies to complete the survey, and determine the level of experience at the time they started the survey. This allowed us to fill cells as surveys were completed depending on their answers to the experience questions. To ensure that our final sample included people from multiple traditions and approximately equal numbers of more and less experienced participants, our survey automatically capped participation based on pre-set categories.

#### Recruitment

For each of the therapy categories, we identified instructors, practitioners, and teachers, a group henceforth characterized as *teachers*. We used listservs suggested by our regional experts to contact members around the world who forwarded the request to their peers. Our e-mail recruitment letter explained the study and said that we were seeking “serious students and experienced teachers” to complete our survey.

We explained why we were interested in somatic awareness, as a key element to many mind-body practices, and provided a link to our website where potential participants could determine if they were eligible. Flyers describing the same information and providing the website address were posted at local Bay Area yoga studios and meditation centers. We asked the teachers contacted individually or via listserv to pass information on to students they thought might want to participate and attached a flyer that they could post for others to see or forward by e-mail.

Once individuals logged onto the website, they were asked to select the mind-body therapy they were most familiar with. Next they were asked whether their experience in that therapy was as a student (i.e. learning the practice or receiving the therapy), teacher, or both. Those who responded “student” were then asked how many hours of formal training/instruction/therapy they had, and those with less than 20 hours were ineligible. Those who responded “teacher” were asked how many years of teaching experience they had with the method.

Based on these responses, the program automatically classified eligible individuals into the two groups of less experienced or highly experienced and created eight categories, for one of two levels of experience for each of four types of therapy. Initial participation caps for the four therapy groups were 100 individuals for meditation/mindfulness, 100 for yoga or Tai Chi, 50 for massage, and 50 for Feldenkraïs, Alexander, breath therapy, or body-oriented psychotherapy. In each therapy group, additional caps were put in place to obtain equal sized groups of less or highly experienced individuals.

### Methods: Analysis Plan

Our analyses aimed to identify a set of scales and items from the pool of 63 items that would provide a good fit to the data. As there is no ‘golden rule’ for determining the number of factors or number of items [89], we took a common-sense approach, seeking a number of subscales that was neither too large nor too small, each comprising not too many items nor too few. Theory dictated that the scales not be independent, but neither did we want them too highly correlated.

#### Item analysis

We began our pruning operation with an inspection of item means, standard deviations, and correlations. From this item analysis we eliminated two highly skewed items.

#### Exploratory and confirmatory factor analyses

Given a theoretical structure that organized items into subscales, the usual next step would have been a confirmatory factor analysis. We are grateful to Steve Gregorich, however, for the suggestion of starting with SAS PROC VARCLUS. Because this procedure has not been widely used, we offer a brief description.

Under the default options we elected, PROC VARCLUS begins with a principal components analysis of the correlation matrix. Starting from a conceptualization of the entire item set as a single cluster, at each step the cluster chosen for splitting is the one with the highest second eigenvalue (provided that that value is greater than 1). Splitting is accomplished by a principal components analysis of the items in that cluster, with a quartimax rotation. The quartimax rotation, maximizing variances of loadings within rows of the structure matrix, tends to produce a general first component, accounting for the maximum amount of variance within the cluster. (The varimax rotation maximizes variances within columns of the matrix, precluding the emergence of a general factor, and favoring interpretation of factors in terms of variables.) Each variable within the cluster is provisionally assigned to whichever of the first two components with which it is most highly correlated. The procedure then tests each variable to see whether assigning it to a different cluster would increase the amount of variance explained; if a variable is reassigned, the components are recomputed before testing the next variable. We did not impose a hierarchical structure on the clustering, so that variables could be assigned to clusters of which they were not originally a part. Cluster components, unlike principal components, are not orthogonal because they are derived from principal components analyses of different subsets of items, rather than from the whole set. The output of PROC VARCLUS looks like what many investigators appear to be seeking with factor analysis: a partition of items into disjoint clusters, listing the squared correlation of each item with its own cluster and with the next closest cluster. The structure matrix looks very much like that from factor analysis; the scoring coefficient matrix differs in having 0s for the items not in a given cluster. Our experience with PROC VARCLUS supports the recommendation and is discussed in more detail below.

As input to PROC VARCLUS, we imputed a covariance matrix using the EM algorithm via SAS PROC MI. For the sample size we conservatively used the smallest *N* for any pair of items, 309.

#### Iterative decision process

The methods applied in the analyses were part of an iterative decision process with elimination of items that performed poorly during various steps of the analyses and decisions about the final number of scales that would provide a good model. Because our initial conceptual framework was hierarchical, with some dimensions of awareness having components or subdimensions, we also had to determine whether to combine any subscales into combined summary scales or retain them as subscales. Conceptually, we had defined 13 possible subscales (see final conceptual framework above) reflecting all sub-dimension in the framework.

Our VARCLUS and CFA analyses were the primary approach to identifying the final number of scales. However, we also examined correlations among all MAIA subscales (high correlations indicated overlapping constructs), item-scale correlations (item-scale correlations corrected for overlap were at least .30), and internal-consistency, and patterns of correlations between each MAIA scale and the validity measures described below (completely redundant patterns of correlation indicated overlapping constructs). The number of potential subscales multiplied by these several ways of exploring their interrelationships precluded specifying hard criteria for each step. We tried to determine if apparently overlapping constructs using one method (e.g., scale-scale correlations) were consistent across approaches (also had similar patterns of correlations with the validity measures). Thus, many of our judgments were based on a synthesis of evidence from all of these analyses.

#### Final confirmatory factor analysis (CFA)

For the final CFA, using Mplus Version 5.21 [90] with the same imputed covariance matrix as with PROC VARCLUS, we were especially guided by the comparative fit index (CFI) and the root mean square error of approximation (RMSEA), as well as modification indices. Following conventional guidelines [91], we required at least two [92] of the following fit indices to fall in the desired range: CFI ≥.90; RMSEA ≤.06; Tucker-Lewis index (TLI) ≥.95; standard root mean square residual (SRMR) ≤.08.

### Results

Recruitment was slower for certain participant categories, and we ultimately were short 2 participants in the experienced yoga/Tai Chi condition, and 3 in the Western somatic therapies. To compensate, we reopened the meditation/mindfulness conditions and recruited another 6 participants. Only participants who fully completed our survey counted toward our cap of 300; we included 47 partially complete cases in our analyses who met our criterion of answering at least half of our 63-item questionnaire. The sample (*N* = 325) was primarily female (79%), Caucasian (about 85%), and well educated (more than half completed graduate school). Mean age was 48 years. Sixty-two percent had more than 10 years of practice. Of the types of therapy they were most familiar with, proportions had been predetermined with most meditation/mindfulness (37%) and yoga/tai chi (32%). Table 1 shows participant characteristics separated by experience level.

As described above, item selection was data-driven and based on an iterative process that allowed for regrouping of items around changing latent variables and dimension constructs.

**Table 1:** Sample Characteristics (total N = 325).

	Less Experienced	Highly Experienced	*p*
Female (*N*, %)	135 (86)	103 (71)	.001 (exact test)
Age (years, mean, SD)	42.2 (11.5)	53.1 (10.5)	<.0001
Race (*N*, %)			.20 (exact test)
White	139 (86)	132 (90)	
Latino	8 (5)	5 (3)	
Asian American	8 (5)	7 (5)	
African American	6 (4)	1 (1)	
Other	0	2 (1)	
Education (*N*, %)			?^2^(2) = 4.42, *p* = .11
No college degree	15 (9)	18 (12)	
College degree	72 (43)	49 (32)	
Graduate degree	81 (48)	88 (57)	
Years experience (mean, SD)			?^2^(3) = 99.48, *p*<.0001
1-4	42 (27)	0 (0)	
5-6	25 (16)	3 (2)	
7-10	34 (22)	15 (10)	
>10	56 (36)	135 (88)	
Primary practice (*N*, %)			?^2^(4) = 5.86, *p* = .21
Meditation/mindfulness	64 (40)	55 (36)	
Yoga/Tai Chi	48 (30)	44 (29)	
Massage	27 (17)	27 (18)	
Body-oriented therapy	15 (9)	11 (7)	
Other somatic therapy	6 (4)	16 (10)	

For an 8-factor model that was identified by the iterative process described above, the CFA (*N = *309; Table 2) showed good model fit according to CFI and RMSEA (Table 3) and acceptable fit according to CFI and TLI. Nine items had modification indices above 10 (eight in the range of 10 to 13.4 and one item 21.5). We also attempted to determine whether a summary score for all 32 items would simplify the measurement of our construct by forcing all items into a single factor model. However, the fit indices showed a predictably poor fit (Table 3). To determine whether all eight factors could support an overall interoceptive awareness construct, we also tested a hierarchical model, with the eight factors as indicators of one overall second-order factor. The fit indices showed a fit to the data almost as good as the first-order CFA. All loadings were significant at *p*<.001 for all three models (Table 3).

**Table 2:** Items and Standardized CFA Loadings.

	Standardized loading	SE
**Noticing**		
1. When I am tense I notice where the tension is located in my body.	.697	.039
2. I notice when I am uncomfortable in my body.	.594	.045
3. I notice where in my body I am comfortable.	.711	.038
4. I notice changes in my breathing, such as whether it slows down or speeds up.	.452	.053
**Not-Distracting**		
5. I do not notice physical tension or discomfort until they become more severe.	.631	.050
6. I distract myself from sensations of discomfort.	.644	.050
7. When I feel pain or discomfort, I try to power through it.	.622	.051
**Not-Worrying**		
8. When I feel physical pain, I become upset.	.629	.049
9. I start to worry that something is wrong if I feel any discomfort.	.724	.046
10. I can notice an unpleasant body sensation without worrying about it.	.577	.051
**Attention Regulation**		
11. I can pay attention to my breath without being distracted by things happening around me.	.589	.041
12. I can maintain awareness of my inner bodily sensations even when there is a lot going on around me.	.766	.027
13. When I am in conversation with someone, I can pay attention to my posture.	.625	.038
14. I can return awareness to my body if I am distracted.	.728	.031
15. I can refocus my attention from thinking to sensing my body.	.758	.028
16. I can maintain awareness of my whole body even when a part of me is in pain or discomfort.	.747	.029
17. I am able to consciously focus on my body as a whole.	.721	.031
**Emotional Awareness**		
18. I notice how my body changes when I am angry.	.518	.045
19. When something is wrong in my life I can feel it in my body.	.534	.044
20. I notice that my body feels different after a peaceful experience.	.817	.024
21. I notice that my breathing becomes free and easy when I feel comfortable.	.809	.025
22. I notice how my body changes when I feel happy/joyful.	.837	.023
**Self-Regulation**		
23. When I feel overwhelmed I can find a calm place inside.	.730	.032
24. When I bring awareness to my body I feel a sense of calm.	.736	.032
25. I can use my breath to reduce tension.	.773	.029
26. When I am caught up in thoughts, I can calm my mind by focusing on my body/breathing.	.735	.032
**Body Listening**		
27. I listen for information from my body about my emotional state.	.761	.030
28. When I am upset, I take time to explore how my body feels.	.769	.030
29. I listen to my body to inform me about what to do.	.822	.026
**Trusting**		
30. I am at home in my body.	.601	.042
31. I feel my body is a safe place.	.831	.028
32. I trust my body sensations.	.817	.029

**Table 3:** Confirmatory Factor Analyses Model Fit Indices.

	?^2^/DOF/*p*	CFI	TLI	RMSEA (CI)	SRMR
1-Factor Model	2126.5/464/.0000	.615	.588	.108 (.103–112)	.088
Hierarchical Model	1035.6/456/.0000	.866	.854	.064 (.059–069)	.067
8-Factor Model	927.3/436/.0000	.886	.870	.060 (.055–066)	.056

*χ* ^2^/DOF/*p*: Chi Square/degrees of freedom/*p* value.

CFI: Comparative Fit Index.

TLI: Tucker-Lewis Index.

RMSEA (CI): Root Mean Square Error of Approximation (95% Confidence Interval).

SRMR: Standard Root Mean Square Residual.

The final MAIA survey consists of 32 items comprising eight scales ranging from 3 to 7 items each. The final scales organized according to the final conceptual framework are presented in Table 4 with their definitions and factor loadings, and Table 5 summarizes the internal-consistency reliability and descriptive statistics of the scales. These scales include items that either duplicate or are similar to items from previously published and developed scales: five items (MAIA items 1, 6, 18, 20, 27) from the SBC [57], one item from the BRS [93] (MAIA item 29), one item from the Mindful Attention Awareness Scale [94] (MAIA item 5) and one item from the Kentucky Inventory of Mindfulness Skills [95] (MAIA item 4).

**Table 4:** Final Multidimensional Conceptual Framework of Body Awareness and Scales.

1) *Awareness of Body Sensations*
** Noticing**: Awareness of uncomfortable, comfortable, and neutral body sensations
2) *Emotional Reaction and Attentional Response to Sensations*
** Not Distracting**: Tendency to ignore or distract oneself from sensations of pain or discomfort
** Not Worrying**: Emotional distress or worry with sensations of pain or discomfort (reversed)
3) *Capacity to Regulate Attention: ability to stay focused when facing numerous sensory stimuli competing for attention*
** Attention Regulation**: Ability to sustain and control attention to body sensation
4) *Awareness of Mind-Body Integration: access to more developed levels of body awareness*
** Emotional Awareness**: Awareness of the connection between body sensations and emotional states
** Self-Regulation**: Ability to regulate psychological distress by attention to body sensations
** Body Listening**: Actively listens to the body for insight
5) *Trusting Body Sensations*
** Trusting**: Experiences one’s body as safe and trustworthy

NOTE: Numbered, italicized concepts are overall dimensions; scale names are bolded.

**Table 5:** Reliability, Item-scale correlations, and Descriptive Statistics for MAIA Scales.

Scale	# of items	Item numbers	Alpha	Range of item-scale correlations	Mean (SD)^a^	Observed range^b^
Noticing	4	1–4	0.69	0.35–0.56	3.94 (.59)	1.50–5.00
Not-Distracting	3	5R, 6R, 7R	0.66	0.45–0.49	3.20 (.87)	0.00–5.00
Not-Worrying	3	8R, 9R, 10	0.67	0.44–0.47	3.27 (.84)	0.67–5.00
Attention regulation	7	11–17	0.87	0.54–0.74	3.79 (.64)	1.67–5.00
Emotional awareness	5	18–22	0.82	0.51–0.72	4.16 (.64)	1.80–5.00
Self-regulation	4	23–26	0.83	0.63–0.70	3.86 (.74)	1.75–5.00
Body listening	3	27–29	0.82	0.64–0.73	3.50 (.87)	0.67–5.00
Trusting	3	30–32	0.79	0.53–0.68	4.13 (.74)	1.00–5.00

a All scales are scored so that a higher score is more positive body awareness; thus Distracting and Worrying are scored so that a high score is less distracting and less worrying.

b Possible range from 0–5.

R = reversed scored items.

The eight final scales reflected five overall dimensions, with up to three subscales representing each dimension. Internal-consistency reliabilities ranged from .66 to .82; unstandardized alphas were over .70 for five of the eight scales. Mean scores tended to be high; on a 0–5 scale, means ranged from a low of 3.20 (Not Distracting) to a high of 4.16 (Emotional Awareness). For some scales, the lowest observed score was well above the minimum; e.g., the minimum observed score for Emotional Awareness was 1.8 and for Self-Regulation, 1.75.

Correlations among the eight scales (Table 6) ranged from.09 to.60 (median .35) indicating independence. The highest correlations were between Body Listening and Emotional Awareness (.60), Noticing and Attention Regulation (.56), and Self-Regulation and Attention Regulation (.55).

**Table 6:** Pearson Product-Moment Correlations among the Eight MAIA Scales.

Scale	Noticing	Distracting	Worrying	Attention regulation	Emotional awareness	Self-regulation	Body listening	Trusting
Noticing	–							
Not-distracting	.26	–						
Not-worrying	.16	.33	–					
Attention regulation	.56	.31	.35	–				
Emotional awareness	.47	.23	.09	.45	–			
Self-regulation	.35	.19	.31	.55	.50	–		
Body listening	.44	.29	.19	.45	.60	.54	–	
Trusting	.38	.32	.31	.50	.34	.52	.44	–

### Changes in Conceptual Framework Resulting from Field Test

Compared to the conceptual framework on which the field test was based (Part 1.7), the final scales reflect all of its five dimensions and most subdimensions.

Dimension 1, Awareness of Body Sensations, stayed intact and was relabeled as Noticing.

For Dimension 2, Emotional Reactions and Attentional Response to a Sensation, two of the four subdimensions were retained in the final scales. Items for Affective Response to Sensations (2A) were not retained, after one item moved to our Not Worrying scale (details below); and we dropped the remaining four items for being pulled in multiple directions, lack of contributing to any single item cluster, or being highly skewed. Dimension 2B, Ignoring or Avoiding Perceptions of Sensations such as by Distracting Oneself, remained intact and was relabeled as Not Distracting, so that higher scores represented more body awareness (less distracting). For 2C, Narrative, Judgmental Awareness, we lost two items due to low factor loadings, and gained one item from the Emotional Reaction to a Body Sensation scale. As the resulting scale items were specific to worrying and no longer adequately represented the original construct, we renamed this scale Not Worrying. We also could not retain our 2D subdimension of Present-Moment Awareness, as four items were dropped due to lack of contributing to any single factor and the remaining item asked to be moved to another factor.

For Dimension 3, Capacity to Regulate Attention, the subdimension Allowing was lost after its three items were dropped due to low factor loadings and lack of clarity. After dropping four items from the remaining subscales due to redundancy and high modification indices, separate subdimensions could not be distinguished in the CFA, resulting in a single merged scale for Attention Regulation, containing seven items.

For Dimension 4, Trusting Body Sensations, we dropped three items that were highly skewed or had low factor loadings, and gained one item from the earlier 5C, Sense of an Embodied Self construct. The remaining items still fit well with the earlier concept, and thus constituted our final Trust scale.

In Dimension 5, Mind-Body Integration, Emotional Awareness (5A) stayed intact through all analyses and lost one item due to lack of factor contribution. Subdimension 5B, Self-Regulation of Emotions, Sensations, and Behavior, lost one item for low factor loadings and split off into two new scales: Body Listening and Self-Regulation. Subdimension 5C, Sense of an Embodied Self, lost two items due to low factor loadings and high modification indices; as the remaining items did not represent a clear construct, we dropped this subdimension.

### Discussion

According to the results from three models of CFA, a conceptual model that includes eight separate dimensions of interoceptive awareness seems to best fit the data sampled in a mind-body therapy-experienced population. The field test data allowed the reduction of the tested item pool from 63 items to 32 items.

The mixed-method process chosen in the development of the MAIA is an attempt to respect the relative newness and abstractness of the construct. As is common in measure development, we conceive of CFA as a *framework* for model development, where different phases may be more exploratory or more confirmatory (example in [96]). Since PROC VARCLUS does not provide good measures of fit, we used CFA, not as a test, or strict confirmation, but for a quantitative assessment of goodness of fit.

The clustering that PROC VARCLUS yielded consistently constituted a good match to our theoretical grouping of items; any differences that emerged were often interpretable in theoretical terms. In addition, the PROC VARCLUS output typically performed well when input to a CFA. In contrast, the initial CFAs done prior to using PROC VARCLUS did not show a good fit and suggested changes by the modification indices that were often not theoretically appealing. We speculate that the superior performance of PROC VARLCUS over exploratory and confirmatory factor analysis may be explained by its use of item clusters, defined by correlations, which may approximate human cognition better than analyses by weights defining straight lines in multivariable space. PROC VARCLUS appears to function effectively as an intermediary between clustering and scale definition by using principal components analysis as a means of clustering items.

The emergence of separate new subdimensions from our original framework may, therefore, be appreciated as improved discrimination of our dimensions based on the focus group participants and on responses from our field test participants. The loss of subdimensions from our initial conceptual framework, particularly of Allowing, which was introduced by the instructor focus group, and the critical distinction of Judgmental, Narrative Awareness from Present-Moment Awareness, may be viewed as limitations of the MAIA and warrant further discussion. Is it possible that our choice of using experienced mind-body instructors as the field test sample population biases the responses to these items in a way that these dimensions are unable to form separate item clusters or factors? We will explore this question in a separate test sample of body awareness-naïve subjects in a subsequent paper. However, two of the original items for Judgmental, Narrative Awareness remained in another dimension; and the largest MAIA dimension, Attention Regulation, with seven items, includes key elements of attention skills that can be viewed as conditional for present moment awareness, a key aspect of mindfulness. For example, the item “I can pay attention to my breath without being distracted by things happening around me” refers to skills of sustained attention and attention control that are elements of mindfulness and have been described as a component through which mindfulness may exert its effects [97]. Similarly, the item “I can refocus my attention from thinking to sensing my body” relates to the ability to switch from narrative to mindful awareness, the precise elements of awareness we had intended to have separate dimensions for. Although we are moderately confident that assessing this critical element is not lost from our instrument, we would like to invite the research community to take part in the further refinement of these conceptual dimensions of interoceptive body awareness.

Our finding that the scales tended to be skewed toward higher levels of body awareness is consistent with the sampling strategy (adults with at least some experience in body awareness therapies). Because an important application is to detect changes over time, it is important to assure that the scales also work in more naïve subjects. In our construct validity analyses, we report descriptive statistics separately for the more and less experienced participants (see Table 7). As seen there, variability increases slightly in the less experienced group. In patients with little experience with mind-body therapies, we expect to observe the full range for all scales and substantially lower means than in this sample (separate publication).

**Table 7:** Mean Differences in MAIA Scales by Level of Experience.

Subscale	Students and less experienced teachers N≥164		Teachers with >5 years of experience N≥155		
	Mean (SD)	Observed range	Mean (SD)	Observed range	*p* (t-test)
Noticing	3.79 (.60)	1.50–5.00	4.09 (.54)	2.00–5.00	<.0001
Not-Distracting	3.13 (.79)	0.67–5.00	3.28 (.93)	0.00–5.00	0.13
Not-Worrying	3.13 (.88)	0.67–4.67	3.42 (.77)	0.67–5.00	0.002
Attention Regulation	3.65 (.68)	1.67–5.00	3.95 (.56)	1.71–5.00	<.0001
Emotional Awareness	4.13 (.68)	1.80–5.00	4.19 (.61)	2.20–5.00	0.38
Self-Regulation	3.79 (.74)	1.75–5.00	3.93 (.72)	1.75–5.00	0.07
Body-Listening	3.41 (.94)	0.67–5.00	3.60 (.78)	1.33–5.00	0.04
Trusting	4.09 (.75)	1.00–5.00	4.17 (.74)	1.67–5.00	0.33

a All scales are scored so that a higher score is more positive body awareness;

b Possible range from 0–5.

## Part 3: Construct Validity: Relationships between Maia and Other Constructs

For new measures such as the MAIA scales, evaluation of correlations with other measures provides the first step in understanding the meaning of the measures. Using the field test sample, we performed two integrated analyses of these relationships: (a) determining if the measures relate to other measures in ways consistent with plausible hypotheses [98], [99], and (b) examining correlations of each MAIA scale across all of the validity measures and interpreting the meaning of each scale in terms of the correlation patterns.

### Methods

To test convergent and discriminant validity, we included in the survey several published measures of constructs related to body awareness. Four types of instruments were chosen for this analysis: (a) aspects of body awareness and mindful attention: Five Factor Mindfulness Questionnaire, Body Consciousness Questionnaire, Body Responsiveness Questionnaire; (b) anxiety as state and trait or as distress in response to bodily symptoms or pain: State-Trait Anxiety Inventory, Anxiety Sensitivity Index, Pain Catastrophizing Scale; (c) dissociation from the body and bodily sensations: Multiscale Dissociation Inventory (MDI [100]) Depersonalization subscale, Body Dissociation subscale of the Scale of Body Connection; and (d) the ability to regulate emotions: Emotional Approach Coping Scales, Difficulties in Emotion Regulation Scale. For some multidimensional measures, we selected a subset of dimensions that would be potentially associated with our scales. Two of these measures (MDI, STAI-S) were extremely skewed in our sample (minimal scores for dissociation and state anxiety) and were dropped from further analysis.

### Measures of Aspects of Mindful Attention and Body Awareness

Because one of our goals was to develop an instrument that can distinguish between an anxiety-driven hypervigilance to body sensations and a present-moment, mindful and accepting attention style to the same body sensations, measures of mindful attention were included to assess convergent validity of this aspect of the new instrument. We describe each measure including the rationale for including it, and report the internal-consistency reliability of the measures in our sample.

#### Five Factor Mindfulness Questionnaire (FFMQ) [101], [102]

The FFMQ, a 39-item, multidimensional self-report scale, is one of the most widely used, well-established measure for mindfulness. Body awareness, although integral to the mindfulness construct [53], [94], [97], [103], has not been assessed as such in mindfulness instruments. Mindfulness extends beyond body awareness to the awareness of exteroceptive stimuli and thoughts. The FFMQ subscales include items assessing the ability to observe body sensations among various other stimuli (Observing; OBS), describe emotions (Describing; DSC), attending to one’s activities of the moment (Acting with Awareness; AWA), and attend to (Nonjudging; NOJ) and accept (Nonreactivity; NOJ) body sensations. FFMQ subscale internal-consistency reliabilities ranged from .76 to .92.

#### Body Consciousness Questionnaire, Private Body Consciousness (PBCS) [104]

The PBCS, a 5-item self-report subscale of the Body Consciousness Questionnaire, assesses sensitivity to internal bodily tensions and the ability to notice sensations such as one’s mouth or throat getting dry, the heart beating, stomach hunger contractions, and changes in body temperature. The PBCS, largely focusing on awareness of inner bodily sensations, thus involves interoceptive skills. Internal-consistency reliability of the PBCS was .74.

#### Body Responsiveness Questionnaire (BRQ) [93]

The BRQ is a 7-item self-report measure consisting of two dimensions that assesses general attitudes towards and responsiveness to bodily sensations. The first dimension, Importance of Interoceptive Awareness, assesses the importance of listening to bodily sensations (LSTN) to enhance self-awareness and guide decision-making. The second dimension, Perceived Disconnection (PD), assesses the perceived lack of integration between psychological and bodily states (reversed scored for total measure). Higher scores on the overall BRQ have been associated with yoga practice and less disordered eating [93] and greater psychological well-being [105]. Body responsiveness also increased among overweight and obese women participating in a mindfulness intervention for stress eating compared to waitlist controls [106]. The internal-consistency reliability of the two BRQ scales was .73 (PD) and .82 (LSTN).

### Measures of Anxiety as State or Trait or as Distress in Response to Bodily Symptoms or Pain

A systematic review of existing body awareness instruments [4] found no validated measures of body awareness that can distinguish between anxiety-related hypervigilance toward pain and a nonjudgmental, meditative, mindful awareness of these sensations. The new measure is designed to discriminate between these. Thus, we included well-established anxiety measures for confirmatory and discriminant validity assessment.

#### Anxiety Sensitivity Index (ASI), Physical Concern subscale (ASI-PC) [107]

Anxiety sensitivity is defined as the fear of anxiety-related bodily sensations based on the belief that these sensations have harmful somatic, social, or psychological consequences. It is seen as a trait, the proneness or enduring tendency to become frightened by anxiety-related sensations [108]. The ASI is a 16-item measure that assesses fear of arousal symptoms by three subscales. The Physical Concerns 7-item subscale (ASI-PC) assesses the tendency to worry when experiencing bodily sensations of quickened respiration or heartbeat, chest constriction, or generalized bodily discomfort. The ASI-PC subscale internal-consistency reliability was .93.

#### Pain Catastrophizing Scale (PCS) [109]

The PCS is a 13-item measure to assess catastrophizing in response to pain sensations, with three subscales: Rumination, the inability to inhibit persisting pain-related thoughts (RUM); Magnification, the concern that the pain will get worse or have a negative outcome (MAG); and Helplessness, worry about pain and the sense of being overwhelmed by it (HLP). Pain catastrophizing overlaps with anxiety [110], but is a more pain-specific, worry-related construct. Internal consistency reliabilities were .70 (MAG), .89 (HLP), and .93 (RUM).

#### State-Trait Anxiety Inventory (STAI) [111]

We used the original 20-item STAI Trait Anxiety scale for convergent validity assessment of the MAIA-Worrying subscale (reverse scored to show tendency *not* to worry). Anxiety measures often include body symptoms or sensations, but it is one of our key assumptions that the interoceptive ability to notice subtle body sensations is distinct from the typical automatic and reactive processes that underlie worry and anxiety. Internal consistency reliability for the STAI-T was .92.

### Measure of Dissociation from the Body

A lack of awareness of or connection to the body is recognized as integral to the *bodily* dissociation process and experience [8], [112]–[114]. Bodily dissociation does not intrinsically indicate pathology, though separation from physical and sensory experience is integral to the construct of pathological dissociation, [115] understood as a mechanism to cope with emotional and physical pain. Bodily dissociation has been shown to be conceptually independent from body awareness, [57] hence our interest in assessing its correlation with the MAIA scales.

#### Scale of Body Connection, Bodily Dissociation Subscale (SBC-BD) [57]

The Bodily Dissociation (BD) subscale of the Scale of Body Connection is an 8-item measure to assess a sense of separation from sensory and emotional experience. Women in treatment for substance use disorder undergoing a body-oriented therapy intervention aimed at increasing body awareness and association improved in scores of bodily dissociation compared with treatment as usual [116]. Significant reductions in bodily dissociation were also demonstrated among women in recovery from sexual trauma in response to body-oriented therapy and massage [117]. The SBC-BD internal-consistency reliability was .76.

### Measures of Ability to Regulate Emotions

Emotions are often experienced in association with sensations within the body, so-called somatic markers, and there is an intimate link between body awareness or interoception and the ability to regulate emotion [26], [27], [118]. An increased awareness of the body’s response to an emotional stimulus is expected to lead to greater awareness of one’s emotions, and, conversely, an awareness of one’s emotions is a precondition for being able to regulate those emotions [97].

#### Emotional Approach Coping Scales, Emotional Processing Subscale (EACS-EPS) [119]

The Emotional Processing subscale (EPS), one of two self-report Emotional Approach Coping subscales, assesses the acknowledgment or exploration of emotion in response to stressful situations. Such exploration, which is integral to emotion regulation [120], is akin to “listening” to the body and is closely tied to accessing and processing emotions. The EACS-EPS internal consistency reliability was .85.

#### Difficulties in Emotion Regulation Scale (DERS) [121]

The DERS has five subscales to assess various aspects of emotion regulation difficulties, including nonacceptance of emotion (NAC), difficulty in engaging in goal-directed behaviors (GLS), impulse control difficulties (IMP), lack of emotional awareness (AWR), limited access to strategies for emotion regulation (STR), and lack of emotional clarity (CLR). Although interoceptive awareness is integral to emotion regulation, attendance to the body is not explicit in measures of emotion regulation. It is thus theoretically important to examine the relationship between emotion regulation and body awareness scales, particularly scales such as the MAIA that explicitly include dimensions specific to emotion regulation. Internal consistency reliabilities for the six DERS subscales ranged from .80 to .90.

### Hypotheses for Correlations between MAIA Scales and Scales of Related Constructs

Because similar measures tend to correlate in general (one can expect modest correlations among most of our measures and the validity measures), we stated our hypotheses in terms of relative magnitudes. To create the hypotheses, two clinical researchers (CP, WM) independently reviewed a matrix of MAIA scales with each of the scales of related constructs. In light of the large number of validity measures for each MAIA scale, they specified hypotheses for a limited *subset* of validity measures in terms of which pairs would be most highly correlated (including the direction of the association), which would be the next most highly correlated, and which would have little or no correlation. The clinicians then met and discussed and resolved any instances in which they disagreed. Thus, for each of the eight MAIA scales, we made from 3 to 11 specific hypotheses across validity measures in terms of the direction and magnitude, i.e. which would be higher, lower, or in the middle range. We then rank-ordered the actual correlations to determine if the measures hypothesized to be most highly correlated were in the top ranks, measures hypothesized to be moderately correlated were mid-rank, and measures hypothesized to have small or no correlation were in the low ranks.

The **MAIA Noticing** scale, assessing the awareness of uncomfortable, comfortable, or neutral body sensations, was expected to be more highly correlated with aspects of mindful attention and body awareness, particularly and most strongly with the FFMQ-OBS. As the Noticing scale assesses the ability to notice and focus on interoceptive stimuli, we also expected a high positive correlation with the PBCS, but lower than with FFMQ-OBS. In contrast, we expected a smaller and negative correlation with STAI-T.

The **MAIA Not Distracting** scale, assessing the tendency *not* to use distraction to cope with discomfort, was expected to have the highest correlation with the FFMQ-NOR. Owing to its behavioral responses of ignoring or powering through sensations of discomfort, we expected a smaller correlation with FFMQ-OBS and the PBCS. We hypothesized that it would be negatively correlated in the middle rank with the BRQ-PD and the SBC-BD. For measures of emotion regulation, we expected a smaller correlation with DERS-GLS.

The **MAIA Not Worrying** scale, assessing the tendency *not* to experience emotional distress with physical discomfort, was expected to have a higher correlation with the FFMQ-NOR. In relation to measures of anxiety, we hypothesized highly negative correlations with the STAI-T, all subscales of the PCS, and the ASI-PC. For measures of emotion regulation, we also expected relatively high correlations with DERS-NAC, GLS, and IMP.

The **MAIA Attention Regulation** scale, assessing the ability to sustain and control attention to body sensations, was expected to be positively and highly correlated with measures of mindful awareness, FFMQ-OBS, FFMQ-AWA, and FFMQ-NOR, the PBCS, and to a lesser degree with the BRQ-PD. We also expected a negative middle rank correlation with PCS-RUM and, to a lesser degree, with DERS-GLS.

The **MAIA Emotional Awareness** scale assesses the ability to attribute specific physical sensations to physiological manifestations of emotions, an internal process involving a more developed interoceptive awareness or meta-awareness that has matured beyond reflexive reactivity with fear and worry about unfamiliar or irritating bodily sensations. Thus, we expected higher correlations with FFMQ-OBS and DERS-AWR and middle rank correlations with FFMQ-DSC and NOR and the EACS-EPS, as well as with a measure of dissociation, SBC-BD. We also expected lower correlations with the anxiety measures of ASI-PC and STAI-T and a smaller negative correlation with the DERS measure for nonacceptance of emotions.

The **MAIA Self-Regulation** scale, assessing the ability to regulate distress by attention to body sensations, was expected to be more highly correlated with mindful attention scales of FFMQ-NOR, BRQ-LSTN, and to a lesser degree with FFMQ-AWA. We expected a middle-rank correlation with SBC-BD. In regard to emotion regulation measures, we expected a middle-rank correlation with the EACS-EPS and DERS scales for GLS, IMP, and STR, and to a lesser degree with DERS-NAC.

The **MAIA** **Body Listening** scale, assessing the tendency to actively listen to the body for insight, was expected to be more highly correlated with the mindful attention measures of FFMQ-OBS, FFMQ-NOR, and the BRQ-LSTN, as well as with the EACS-EPS and, negatively, the DERS-AWR. We expected middle-rank negative correlations with SBC-BD dissociation and emotion regulation measures of DERS-GLS and IMP, as well as a smaller negative correlation with DERS-STR.

The **MAIA Trusting** scale, assessing the experience of one’s body as safe and trustworthy, was expected to show a high positive correlation with the BRQ-LSTN. For anxiety measures we expected higher negative correlations with the ASI-PC and PCS-HLP and middle-rank negative correlations with PCS-MAG and STAI-T. For a dissociation measure, the SBC-BD, we expected a middle-rank negative correlation, as well as for BRQ-PD and the emotion regulation aspect of the DERS-NAC.

### Examining Patterns of All Correlations

In addition to testing specific hypotheses about the relationships, we were able to build a knowledge base about the meaning of the measures based on the pattern across all measures irrespective of a priori hypotheses. This second approach has previously been taken in exploring the construct validity of the Multidimensional Experiential Avoidance Questionnaire [122], as well as in testing the construct validity of the Medical Outcomes Study (MOS) measures of functioning and well-being [123]. In presenting the results we are integrating both approaches.

### Results and Discussion

As noted above, the internal-consistency reliability of each of the 21 validity measures ranged from .70 to .93 (median .85). Table 8 presents the correlations of each MAIA scale with the 21 subscales included in the 9 validity instruments. This table also shows the six correlations (out of 63) for which the hypothesized strength ranks were not confirmed.

**Table 8:** Correlations between MAIA Scales and Validity Measures.

	Measures of Body Awareness/Mindful Awareness								Measures of Anxiety					Measure of Dissociation	Measures of Emotion Regulation						
Validity Scales MAIA Scales	FFMQ					PBCS	BRQ		AASI-PC	PCS			STAI-T	SBC-BD	EEACS-EPS	DERS					
	OBS	DSC	AWA	NOJ	NOR		PD	LSTN		RRUM	MAG	HLP				NAC	GLS	IMP	AWR	STR	CLR
Noticing	.*53*	.20	.38	.19	.34	.40	−.31	.46	−.19	−.27	−.20	−.23	−.33	−.30	.25	−.26	−.22	−.24	−.36	−.27	−.39
Not-Distracting	.22	.17	**.** ***41***	.36	.30	.20	−.38	.31	−.18	−.24	−.17	−.21	−.35	−.32^a^	.15	−.33^c^	−.28	−.27	−.24^c^	−.32	−.26
Not-Worrying	.16	.13	**.44**	**.38**	.47	.01	−.35	.19	−**.36**	−**.** ***46***	−**.40**	−**.41**	−**.** ***46***	−.27	.11	−**.35**	−**.44**	−**.35**	−.11	−**.39**	−.16
Attention regulation	**.** ***55***	**.38**	.42	.28	**.53**	**.43**	−.33	.48	−.31	−.30	−.19	−.19	−.38	−**.41**	.33	−.24	−.23	−.30	−.41	−.25	−**.42**
Emotional awareness	.*50*	.30	.27	.14	.27	.32	−.24	.48	−.12	−.25	−.15	−.18	−.19	−.33	.34	−.18	−.13	−.17	−.44	−.19	−.38
Self-regulation	.*46*	.18	.36	.22	.45	.29	−.27	.45	−.20	−.34	−.32	−.28	−**.** ***46***	−.27	.27	−.20	−.26	−.27	−.38	−.28	−.28
Body listening	.50^c^	.29	.29	.21	.41	.33	−.33	**.** ***64*** b	−.18	−.34	−.24	−.25	−.29	−.34	**.43**	−.21	−.21	−.22	−**.54**	−.22^c^	−.36
Trusting	.42	.27	.36	.28	.37	.28	−**.40**	.*53*	−.23^c^	−.31	−.24	−.30^c^	−**.46**	−.39	.21	−.22	−.22	−.30	−.39	−.28	−.36

Correlations >.14 are significant at p<.01; correlations >.18 at p<.001; correlations >.21 at p<.0001.

Bolded are the highest correlations in each column, italicized are the highest correlations in each row.

Validity measures:

FFMQ - Five Facet Mindfulness Questionnaire (OBS-Observing, DSC-Describing, AWA-Acting with Awareness, NOJ–Nonjudging, NOR-Non-Reactivity).

PBCS - Private Body Consciousness Scale.

BRQ - Body Responsiveness Questionnaire (PD-Perceived Disconnection, LSTN-Listening to bodily sensations).

ASI-PC - Anxiety Sensitivity Index – Physical Concern.

PCS - Pain Catastrophizing Scale (RUM-Rumination, MAG-Magnification, HLP-Helplessness).

STAI-T - Trait Anxiety Inventory.

SBC-BD - Scale of Body Connection – Bodily Dissociation.

EACS - Emotional Approach Coping Scales – Emotional Processing.

DERS - Difficulties in Emotion Regulation Scale (NAC-Non-acceptance of emotion, GLS-Difficulty engaging in goal-directed behaviors, IMP-Impulse control difficulties, AWR-Lack of emotional awareness, STR-Limited access to strategies for emotion regulation, CLR-Lack of emotional clarity).

a −27 if overlapping item omitted.

b 60 if overlapping item omitted.

c six correlation did not confirm a priori hypothesized rank order.

In the following section, we report the results organized by the eight MAIA scales and further explore the inferred meaning of the measures. We add a brief discussion for each scale to obviate going forth and back between results and subsequent discussion sections.

#### Noticing

All three hypotheses were confirmed. Awareness of uncomfortable, comfortable, or neutral body sensations appears to mean a high capacity to observe with mindfulness (FFMQ-OBS), experience interoceptive stimuli (PBCS), and, not hypothesized, mind-body listening (BRQ-LSTN). As all correlations with measures of anxiety and worry are below .30 (in absolute numbers; maximum .27), with the exception of −.33 for trait anxiety (STAI-T), it appears that noticing body sensations, the more basic, sensory aspect of body awareness, is not particularly strongly related to trait anxiety, at least in mind-body practitioners.

The strong correlations between this MAIA scale and scales of basic perception of body sensations in mindful awareness measures make intuitive sense. Although not hypothesized, the correlation with the BRQ-LSTN is in line with these results. The lack of high correlation between noticing body sensations and trait anxiety scores for mind-body instructors is particularly remarkable, as it is consistent with findings from prior studies that anxious individuals may have heightened vigilance toward body sensations but do not exhibit an increased accuracy of these [124]. This finding confirms our position that at least the basic noticing aspect of the awareness of body sensations can be separated from anxiety; the MAIA-Noticing scale cannot serve as a proxy measure of anxiety. This result, however, may be specific to mind-body practitioners, and we would not necessarily expect it to be confirmed in a mind-body therapy-naïve population.

#### Not distracting

Six of our eight hypotheses were confirmed. At least in practitioners of mind-body approaches, the tendency *not* to ignore or distract oneself from sensations of pain or discomfort appears to mean having a good awareness of when mind-body connection is lacking (BRQ-PD) and, although not hypothesized, having a high awareness of how emotions affect one’s behavior (FFMQ-AWA). As expected, not distracting oneself from negative sensations is moderately correlated with FFMQ-NOR and minimally correlated to FFMQ-OBS. A moderate correlation was confirmed with bodily dissociation as measured by the SBC-BD. This quality is rather distinct from anxiety sensitivity (ASI), the perception of interoceptive stimuli (PBCS), the tendency to magnify negative sensations (PCS-MAG) and the acknowledgment or exploration of emotions in response to stressful situations (EACS) (all correlations lower than .20). Trait anxiety (STAI-T) was found to be moderately correlated with ignoring and distracting oneself from negatively appraised body sensations as the strongest (−.35) correlation with any of the anxiety measures. In regard to measures of emotion regulation, we found smaller correlations with DERS lack of emotional awareness and engaging in goal-directed behavior and a higher than expected correlation with DERS nonacceptance of emotions.

It makes sense to ignore or distract oneself from emotion-related physical sensations when one does not accept a negative emotion. The moderate correlation with trait anxiety supports the notion that trait anxiety may be associated with a coping style that ignores unpleasant body sensations, whereas mindful awareness and nonreactivity (FFMQ) show almost symmetric, opposite correlations. The moderate correlation with bodily dissociation supports the idea that ignoring or distracting behaviors and bodily dissociation are conceptually linked, a result important for better understanding and treatment of pain and physical discomfort. These findings underscore the important role of *not* distracting oneself from body sensations and the utility of this MAIA scale within the body awareness assessment.

#### Not worrying

All nine hypotheses were confirmed. The tendency *not* to experience emotional distress or worry with sensations of pain or discomfort appears to mean, at least in mind-body practitioners, to accept (negative) body sensations (FFMQ-NOR) and, unexpectedly, to be aware of how emotions affect one’s behavior (FFMQ-AWA). Moderate correlations were seen with four measures of emotion regulation: At least in mind-body practitioners, less worrying appears to mean less difficulty in engaging in goal-directed behaviors (DERS-GLS), increased acceptance of emotions (DERS-NAC), increased access to strategies of emotion regulation (DERS-STR), and fewer difficulties in impulse control (DERS-IMP). It also means not to catastrophize pain sensations (all three PCS scales ≥.40), to be low on trait anxiety (STAI-T), and not to worry when experiencing bodily sensations of quickened respiration or heartbeat, chest constriction, or generalized bodily discomfort (ASI-PC). Interestingly, this latent variable or construct is distinct (*r* = .01) from sensitivity to internal bodily tensions and the ability to notice sensations such as one’s mouth or throat getting dry, the heart beating, hunger contractions, and changes in body temperature (PBCS); it is also rather distinct (all *r*s <.20) from the ability to describe emotions (FFMQ-DSC), emotional awareness (DERS-AWR), emotional clarity (DERS-CLR), and an acknowledgment or exploration of emotion in response to stressful situations (EPS).

A stronger tendency *not* to experience emotional distress or worry with sensations of pain or discomfort is consistent with having fewer difficulties in emotion regulation (four of six DERS subscales ≥.35). As expected, this MAIA scale is the one with the strongest correlations with all measures of anxiety. It is possible that the consistently small correlation with emotional awareness and clarity on the DERS, and exploration of emotion on the EPS, means that engagement in these emotional exploratory processes happens regardless of worry in response to bodily discomfort among mind-body practitioners. With these exceptions, this scale has the strongest correlations with measures of emotion regulation: Not worrying when perceiving pain or discomfort may be a condition or a consequence of emotion regulation.

#### Attention regulation

All seven hypotheses were confirmed. The ability to sustain and control attention to body sensations appears to mean a high capacity for mindful observation, awareness, and nonreactivity (FFMA-OBS, AWA, and NOR), awareness of interoceptive indicators (PBCS), and (not hypothesized) valuing the importance of listening to body sensations (BRQ-LSTN). This ability is also moderately correlated with an acknowledgment or exploration of emotion in response to stressful situations (EPS). At least in mind-body practitioners, it also means not to separate oneself from sensory and emotional experience (SBC-BD), and not to lack emotional awareness or clarity (DERS-AWR and CLR) (not hypothesized). It may additionally mean the absence of rumination, but it is clearly distinct from other aspects of catastrophizing (PCS-MAG and HLP).

Of all MAIA scales, Attention Regulation, defined as the ability to sustain and control attention to body sensations, shows the strongest correlations with the FFMQ-OBS and NOR subscales. Skills in attention regulation are a precondition for the capacity to be nonreactive and accepting of body sensations, key elements of more general mindfulness [97]. This scale appears to measure skills related to but distinguishable from aspects of mindfulness captured by the FFMQ. The highest correlations with PCS rumination, anxiety sensitivity, and trait anxiety are only moderate (between .30 and .38), confirming that this scale measures aspects of body awareness independent from anxiety. The moderate correlations with measures of emotional connection, awareness, and clarity suggest that the ability to sustain and control awareness in the body may go along with emotional attunement.

#### Emotional awareness

All nine hypotheses were confirmed. The awareness of the connection between body sensations and emotional states means strong skills in mindful observation (FFMQ-OBS), valuing the importance of listening to body sensations (BRQ-LSTN) and, as expected, a lack of difficulties in emotional awareness and clarity as measured by the DERS AWR and CLR scales. Although anxiety is an important emotion, being aware of the connection between body sensations and emotional states is, as expected, clearly distinct from anxiety as measured by all included anxiety measures, most clearly with anxiety sensitivity (ASI) and trait anxiety (STAI-T). It is also distinct from several aspects of emotion regulation (DERS), namely acceptance of emotion (NAC), engaging in goal-directed behaviors (GLS), impulse control (IMP), and access to strategies for emotion regulation (STR). Moderate correlations were found with FFMQ ability to describe emotions and nonreactivity, awareness of body sensation assessed by PBCS, EACS acknowledgment or exploration of emotion in response to stressful situations, and negatively with body dissociation (SBC-BD).

The awareness of body sensations as being associated with emotions has the highest correlations with two scales of mindful attention and, interestingly, the lowest correlations with measures of anxiety. The specific pattern of correlations with emotion regulation scales is noteworthy: The MAIA scale for awareness of physical symptoms as related to emotions is mostly concordant with measures for acknowledging or exploring emotions in response to stressful situations and with DERS scales of perception, such as having fewer difficulties with emotional awareness and clarity; but it is distinct from DERS scales assessing coping behavior and behavioral outcomes, such as nonacceptance of emotions, difficulty in engaging in goal-directed behaviors, and impulse control difficulties.

#### Self-Regulation

All nine hypotheses were confirmed. As expected, a strong ability to regulate distress by attention to body sensations appears to mean high skills in mindful nonreactivity (FFMQ-NOR) and valuing the importance of body-listening (BRQ-LSTN), in individuals practicing mind-body approaches. Unexpectedly, it is also positively correlated relatively highly with mindful observation (FFMQ OBS), highly negatively with trait anxiety, and moderately with fewer difficulties in emotional awareness (DERS-AWR). It is moderately correlated with skills in the awareness of how emotions affect one’s behavior (FFMQ-AWA), and little correlated with a lack of difficulties (DERS) in goal-directed behaviors (GLS), impulse control (IMP), and strategies for emotion regulation (STR), with exploring emotions in response to stressful situations (EACS), and with all scales of catastrophizing (PCS). It shows a low negative correlation with SBC-Body-Dissociation.

The MAIA scale that measures the ability to regulate distress by attention to body sensations has its strongest concordance with scales of mindful observation, listening to body sensations, and nonreactivity, and clearly less concordance with scales of emotion regulation that do not explicitly assess emotion regulation by attention to body sensations. That a similarly high concordance with the STAI-T was found is no surprise: The body awareness-based self-regulation skills measured with this MAIA scale may be associated with less trait anxiety.

#### Body listening

Eight of ten hypotheses were confirmed. The tendency to actively listen to the body for insight means valuing body-listening skills (BRQ-LSTN), having high skills in mindful observation (correlation stronger than expected) and nonreactivity (FFMQ), and lacking difficulties with emotional awareness (DERS AWR). It is moderately correlated with not ruminating (PCS-RUM), acknowledging or exploring one’s emotions in response to stressful situations (EPS), and body dissociation (SBC-BD). It has little relationship to anxiety sensitivity (ASI).

The strong concordance of this measure with the BRQ scale for the importance of listening to the body is no surprise, particularly as one item is virtually identical between the two scales. A .50 correlation with the FFMQ-OBS scale shows that both scales measure related constructs. Whereas this MAIA scale is exclusively interoceptive, the FFMQ assesses mindful attention to any perceivable stimulus, including thoughts and exteroceptive input. Actively listening to the body for insight was highly (.54) correlated with fewer difficulties with emotional awareness. Interestingly, this was the strongest correlation of any DERS scale with any MAIA scale in our mind-body-experienced field test sample, stronger than the correlation of any other emotion regulation measure with MAIA Emotional Awareness and Self-Regulation. It confirms the construct validity of this scale for our field test sample, though not necessarily in less body-aware individuals.

#### Trusting

Six of eight hypotheses were confirmed. Frequently experiencing one’s body as safe and trustworthy means having strong body-listening skills (BRQ-LSTN) and being relatively free of trait anxiety (STAI-T). Other anxiety measures (ASI-PC, PCS) showed low to moderate correlations. Moderate correlations were found with other mindful awareness measures: with mindful observation (FFMQ-OBS), being aware of how emotions affect one’s behavior, and nonreactivity (FFMQ-AWA, NOR), having little difficulty with emotional awareness or clarity (DERS-AWR and CLR), and not being dissociated from one’s body (SBC-BD).

Correlations with anxiety measures were only moderate, confirming that the body awareness-related Trust scale measures an independent construct that is not simply the inverse of anxiety. The somewhat unexpectedly high (.42) correlation with the FFMQ-OBS scale may be specific to people with mind-body experience.

In summary, 57 of the 63 hypothesized correlations (90%) were found to be rank ordered as expected, confirming the vast majority of MAIA convergent and discriminant hypotheses. The direction of the expected correlation was confirmed in all cases.

### Discussion: Overall Patterns of Relations between Body Awareness and Other Constructs

We conclude this part by discussing our findings for the MAIA scales in the light of four related constructs: mindfulness, anxiety and worry, dissociation, and emotion regulation.

#### Aspects of mindful attention and body awareness

The results indicate that MAIA scales were most highly and positively correlated with aspects of mindful attention and body awareness, particularly strong with the FFMQ-OBS scale, and BRQ-LSTN. This is an expected result given that these validity measures are most similar in construct to the MAIA, to the fundamental aspect of body awareness assessed across the MAIA scales, the ability to notice body sensations. The strong focus of the MAIA on assessing the ability to respond positively to body sensations and experience of the body is confirmed by the BRQ, the only validity measure that focuses specifically on aspects of awareness and response to body sensation. Both scales show moderate to high correlations with six of the eight MAIA scales and support the construct validity of the MAIA. MAIA Not Distracting and Not Worrying show little or no correlation with these two related scales, with Not Distracting being more similar to the FFMQ-AWA and Not Worrying to FFMQ-NOR. The discriminant validity of the MAIA with respect to these measures is supported by the large majority of the correlations being less than .40 (42 out of 64 possible correlations), and approximately two fifths of the correlations being less than .30 (24 out of 64). Whereas the five FFMQ scales each find their highest correlations with only two MAIA scales (Attention Regulation and Not Worrying), the BDQ finds its highest correlation with Body Listening and Trust, suggesting that these MAIA scales assess aspects that are related but distinguishable from each other. The close relationship between the MAIA parameters and the FFMQ-OBS scale in individuals that have had exposure to mind-body approaches may be explained by the following hypothesis: Although the FFMQ-Observe scale does not differentiate between awareness towards interoceptive, exteroceptive, or cognitive stimuli, mind-body therapy-experienced individuals may have learned skills in interoceptive awareness as much as in other objects of mindful observation. This hypothesis needs to be tested in a subsequent study with a separate sample of less experienced individuals.

#### Anxiety and distress in response to bodily symptoms or pain

The results suggest a distinct split in level of relationship between the MAIA and measures of anxiety or worry. Of the three measures chosen to examine aspects of anxiety and distress in response to bodily sensations, only trait anxiety resulted in more than one negative correlation with MAIA scales above .40. Negative correlations above .40 between MAIA and other anxiety/worry measures were with the Worrying MAIA scale only. Three of the MAIA scales, Not Worrying, Self-Regulation, and Trust, were moderately or highly negatively (greater than −.40) correlated with the STAI-T. It appears from these findings that trait anxiety is associated with aspects of body awareness, as would be expected given the inverse relationship between anxiety intensity and the ability to respond positively to sensations in and experience of the body among individuals with anxiety [37]. In contrast, with the exception of the Not-Worrying scale, the MAIA scales appear to have little overlap with assessments specific to fear of arousal or catastrophizing, indicating the discriminant validity of all other MAIA scales in relation to these measures for this sample of mind-body practitioners. The result that trait anxiety is as much positively correlated with worrying as it is negatively correlated with Trust and Self-Regulation underscores the ability of the MAIA scales to distinguish between anxiety-driven and mindful modes of body awareness, and strongly distinguishes the new MAIA scale from older measures using awareness of bodily symptoms as a proxy for anxiety.

#### Dissociation from the body

The SBC Bodily Dissociation scale showed moderate-level correlations with all MAIA scales. The previously shown lack of correlation between body awareness and bodily dissociation [57] did not apply here presumably because of the BD focus on emotion and the overlap with this aspect of awareness on the MAIA. Bodily dissociation inhibits the ability to sustain or control awareness in the body, and to experience trust in the body; thus it is not surprising that the MAIA Attention Regulation scale was the aspect of body awareness most strongly (−.41) and negatively correlated with BD, followed by Trust (−.39).

#### Ability to regulate emotions

The overall findings indicate a moderate relationship between the MAIA and emotion regulation as measured by the EACS and the DERS. Of the multiple emotion regulation subscales examined, only the DERS-AWR is designed to specifically assess awareness of emotion (or the lack thereof), with awareness of emotion-related body sensations being a key aspect of body awareness. The high negative correlations (>.40) between the MAIA scales of Attention Regulation, Emotional Awareness, and, strongest, Body-Listening (.54) and the DERS-AWR subscale support the construct validity for this integral aspect of the MAIA body awareness construct. The correlations between the MAIA and other emotion regulation subscales indicate that these subscales assess aspects of emotion regulation that, while generally moderately correlated, are less strongly associated with body awareness as represented on the MAIA. Although listening to the body was associated with emotional awareness, and attention regulation with emotional clarity, in general a style of emotion regulation that is based on body awareness (as assessed by the Self-Regulation MAIA scale) appears to be related to, but clearly distinct from, emotion regulation skills assessed by EACS and DERS.

## Part 4: Construct Validity: Differences between Known Groups

For further evaluating construct validity of the MAIA, we examined whether the MAIA scales show differences in body awareness between known groups that are theoretically expected to be different.

### Methods

Using *t* tests for data collected in the field test population, we compared mean scores on the eight MAIA scales between the two groups of more and less highly experienced participants according to the sampling design reported in Part 2.

### Results

We had a total of 165 less experienced and 157 highly experienced field test respondents (Table 2). The two groups did not differ in ethnicity or education. The highly experienced group had a higher percentage of male participants (29% versus 14%) and was on average 11 years older.

As presented in Table 7, the highly experienced group had significantly higher mean scores on four of the eight MAIA scales. All differences were in the expected direction. The largest (most significant) differences were for the Noticing and Attention Regulation scales.

### Discussion

The results support the scales’ ability to distinguish between known groups. As the less experienced group also included a group of nonteaching students with more than 5 or 10 years of practice (73% and 36%, respectively), as well as junior teachers, we did not expect differences as large as one would expect between beginners and teachers. Nevertheless, differences were highly significant for key elements of mind-body training that have been claimed to improve with more practice: Highly experienced participants were more often aware of their body sensations, less frequently worried about sensations of pain and discomfort, better able to regulate their attention focus, and listened more frequently to the body for insight. Also, results were marginally significant for highly experienced respondents being more often able to regulate psychological distress by attention to body sensations.

For the tendency to ignore or distract oneself from sensations of pain or discomfort, we reported elsewhere some preliminary results showing that common primary care patients with past or present low back pain significantly more often (<.001) distract themselves and ignore their pain or discomfort than our field test participants practicing a mind-body approach (separate manuscript in preparation). [125] It is possible that a moderate amount of experience may suffice to improve emotional awareness and more frequently experience the body as safe and trustworthy, and that longer practice duration does not further enhance these aspects of body awareness; this hypothesis remains to be tested by comparing mind-body therapy-naïve with experienced individuals.

## Part 5: Construct Validity: Incremental Validity for the Maia Scales

To examine whether a multidimensional assessment of body awareness provides incremental validity in explaining the relationship between body awareness and clinical outcomes, we examined the relationship between scores on the MAIA scales and the STAI-T. As described earlier, body awareness may be associated with more or less anxiety depending on whether it is conceptualized as an adaptive or maladaptive form of attention. Thus, it is also of theoretical interest to examine the extent to which different aspects of body awareness are associated with anxiety.

### Method

For these purposes, we entered all of the MAIA scales simultaneously into a linear regression model to predict anxiety scores. While each MAIA scale bore a significant bivariate correlation with anxiety, a substantial contribution by a multiplicity of MAIA scales provides evidence of incremental validity, and suggests that consideration of multiple aspects of body awareness is helpful in understanding the relationship between body awareness and anxiety.

### Results

Results of this analysis can be seen in Table 9. Noticing, Not Distracting, Not Worrying, Self-Regulation, and Trusting were significant, and Emotional Awareness marginally so, indicating that each scale accounts for a portion of the variance in anxiety not accounted for by the others.

**Table 9:** Regression Analysis Showing Incremental Validity of MAIA Scales in the Prediction of Trait Anxiety.

	*B*	SE	β	*t*	*p*
Noticing	−2.45	0.87	−0.16	−2.82	0.005
Not Ignoring	−1.56	0.53	−0.15	−2.96	0.003
Not Worrying	−2.77	0.54	−0.26	−5.17	<0.0001
Attention Regulation	0.52	0.87	0.04	0.60	0.55
Emotional Awareness	1.50	0.83	0.11	1.81	0.07
Self-Regulation	−3.46	0.74	−0.29	−4.67	<0.0001
Listening	0.30	0.62	0.03	0.48	0.63
Trusting	−2.27	0.66	−0.19	−3.42	0.0001

Note: *R* ^2^ for model = .41.

### Discussion

It appears that both maladaptive aspects of body awareness (including ignoring and worrying about body sensations) and adaptive aspects (including noticing body sensations, having the ability to reduce distress by attending to bodily sensations, and experiencing the body as safe and trustworthy) are important in understanding the relationship between body awareness and anxiety. Interestingly, while Emotional Awareness is related negatively to anxiety in a bivariate correlation, in the regression model greater emotional awareness was related to greater anxiety, although the effect was of marginal significance. To understand this unexpected suppressor effect, separate regressions were run with Emotional Awareness and each of the other MAIA scales to predict anxiety (results not shown). In all cases, the relationship between Emotional Awareness and trait anxiety remained negative, except for Self-Regulation, in which case the coefficient became positive. These findings indicate that Emotional Awareness shares variance with Self-Regulation, and, once the shared variance is removed, the relation with anxiety becomes positive, suggesting that aspects of emotional awareness are both positively and negatively related to anxiety. One interpretation is that mere awareness of how body sensations correspond to emotional states, without the ability to use awareness of those sensations to reduce distress, could increase anxiety. This distinction could help to clarify the dual theories of how body awareness affects anxiety. As a word of caution, this incremental validity test was performed among experienced mind-body practitioners, and not among those with an anxiety disorder, thus these results may have limited generalizability and need confirmation in a separate sample.

In general, results of the incremental validity regression suggest that a multidimensional assessment of body awareness may be valuable in understanding clinical outcomes. As the MAIA is meant to be useful for understanding a multitude of medical and psychological conditions, future research will need to be conducted to determine the incremental validity for other outcomes.

## Part 6: Overall Discussion

This paper describes the development and preliminary validation of the MAIA. The CFA confirmed eight scales reflecting distinct but related dimensions of interoceptive body awareness. The results indicated adequate goodness-of-fit indices, supporting the construct validity of the MAIA scales. As a word of caution, we need to emphasize that this preliminary validation requires confirmation in a separate sample.

The study also generally demonstrated the internal consistency reliability of the scales. The three alphas slightly below .70 may be of some concern. We decided to accept alphas greater than 0.65 for three- to four-item scales, in an effort to reduce questionnaire burden. Because scales with as few as three items are more sensitive than longer scales to the similarity of the questions, it may be helpful for future research to explore the addition of items to enhance these particular scales. It should also be borne in mind that correlations between MAIA scales and scales of related constructs are theoretically limited by the reliability of each scale. For a short scale with an alpha, for example, of 0.69, a correlation of .53 with a scale of a related construct is near its theoretical maximum.

To explore the construct validity of the MAIA scales, we took three broad approaches. First, construct validity was assessed by correlations between MAIA scales and scales of related constructs. Expected correlations were specified and largely confirmed by the data from a field test in a mind-body therapy-experienced population sample. The meaning of the new MAIA scales was explored by examining the resulting correlations irrespective of the a priori hypotheses. Correlation patterns demonstrated differential relationships between the MAIA and similar scales of body awareness, mindfulness, and emotion regulation, providing support for the MAIA scales as both a distinct and multidimensional conceptualization of body awareness. Second, construct validity was also shown by differences in MAIA scale scores between less and more highly experienced mind-body therapy practicing individuals. And third, regression analysis showed incremental validity for multiple dimensions of the MAIA scale in understanding anxiety as an example of a clinical outcome.

Any psychometric assessment of a measure for a construct that involves skills and new language learned during a practice or intervention necessarily encounters the difficulty that the understanding of questionnaire items may change during this learning process. This difficulty was encountered and well-described in the development of the FFMQ. For the FFMQ, the problem with the “observe” facet, created by factor analysis and *not* well-validated in individuals without meditative experience, was described as a shortcoming in the original publication [101]. The authors addressed this issue in a later publication after the questionnaire was submitted to individuals with meditative experience [102]; however, they admittedly were unable to resolve it [126]. For our study, we decided to do the analysis in a reverse order, to generate the factor structure and subscales first in a sample of experienced individuals with a more developed understanding of the questions, and then test the questionnaire in individuals without that experience. This way we expected to obtain a selection of items with greater face validity for the qualities we expect to improve in individuals undergoing these approaches. We are well aware that reversing the order of analysis does not solve the more general problem of different understanding of the items in different populations. However, we attempted to avoid in part the problem the FFMQ team encountered and gave preference to face validity of items in more experienced individuals in order to ensure that the new insights individuals gain during their exposure to these approaches (described in the report of the focus groups [87]) are well captured.

Several limitations need to be considered. First, as we particularly wanted to develop a measure that would capture potential changes in body awareness over time as people learn and practice therapies that claim to enhance body awareness, we were interested in correlations between established validity measures and scores on our MAIA scales for individuals who had exposure to these approaches. Their exposure to mind-body therapies likely affected the way the questions were understood [125]. However, the language that was used in creating the items was cognitively tested with a sample that included individuals without any such exposure. The psychometric performance of the MAIA requires assessment in a population without such exposure, which will be the subject of a separate study.

Second, the current paper presents the psychometric evaluation of the MAIA scales in a single population. The observed correlational patterns could be idiosyncratic to the present sample and need psychometric exploration in additional samples.

Third, the current paper presents the results of the field test in a healthy population. We tested the construct validity of the new MAIA scales with a large number of measures of related constructs measured concurrently, and used the STAI-T trait anxiety scale to determine the ability of the MAIA scales to independently predict subclinical levels of this important outcome. Future research could explore validity further by examining its associations to more clearly defined clinical conditions, such as chronic pain, addiction, or eating or anxiety disorders.

Fourth, we started the field test with 63 items and dismissed many of the original items during our item selection process. This resulted in a data-driven modification of our original construct and the loss of several aspects of body awareness discussed in Part 2. Owing to poor psychometric performance in the field test, we lost the dimensions of Allowing, Judgmental, Narrative Awareness, and Present-Moment Awareness. These aspects should be considered in future editions of the instrument. However, as we argued above, the loss of these dimensions may in part be compensated by the 7-item scale of Attention Regulation. The Attention Regulation scale showed the largest difference between more and less highly experienced mind-body practitioners. We would expect that this is a particularly important skill that continues to be strengthened with increasing training in mind-body approaches.

The fundamental limitation of the MAIA is that it is self-report. One consequence is that it is largely capturing intra- rather than interindividual variability, since respondents have so little information about other people’s body awareness; of necessity they will be reporting deviations from their own baseline. This is of course a limitation of all psychological self-report scales. Indeed, given that all of our validity measures themselves contain the same validation gap, our imposing validity matrix is still a monomethod matrix. But, as an assessment of awareness, the MAIA adds another significant difficulty: the special challenge of reporting something of which we may be unaware. Hence it will be especially important in the case of the MAIA to seek validation by other, more “objective,” means, such as behavioral measures (heart rate and airway resistance detection tasks) or fMRI changes in brain activities.

A major strength of the MAIA instrument is its multidimensionality. Whereas prior instruments were unable to distinguish between beneficial and maladaptive aspects of interoceptive body awareness, the new scales allow a more differentiated assessment of essential psychological aspects of the perception and evaluation of body sensations. This instrument has the potential to further our understanding of psychosomatic mechanisms of action for a variety of mind-body interventions. In terms of treatment for clinical conditions, such as anxiety, understanding which aspects of body awareness are related to clinical outcomes could help inform the design of mind-body therapies aimed at treating those conditions. Future research on mind-body interventions could also use a multidimensional assessment of body awareness to understand which aspects of body awareness contribute to improvements in clinical outcomes.

In summary, the systematic development of a new self-report instrument for interoceptive awareness resulted in the MAIA, a 32-item multidimensional instrument with eight separately scored scales. A field test in a sample of individuals familiar with a variety of mind-body therapies provided acceptable psychometric results and support for construct validity. It needs to be determined how the MAIA scales function in a population that has not been exposed to these therapies, and whether the scales are sensitive to changes in longitudinal studies. Further cross-sectional and longitudinal validity data using the MAIA in different populations are currently being collected and analyzed. As the constructs of interoception and body awareness warrant a multidisciplinary team approach for their operationalization, we would like to invite other researchers across disciplines for broad cooperation in the refinement and further development of valid measures of interoceptive body awareness. We view the MAIA as an appropriate starting point in this important research field.

## Supporting Information

Supporting Information S1 The Multidimensional Assessment of Interoceptive Awareness (MAIA), questionnaire with scoring instructions.
(DOCX)
